# Supplementary material for: Impacts of burn severity on short-term postfire vegetation recovery, surface albedo, and land surface temperature in California ecoregions
Source: PLoS One. 2022 Nov 3;17(11):e0274428. doi: 10.1371/journal.pone.0274428 (PMC9632817; doi:10.1371/journal.pone.0274428)
Supplement: Attachment — Submitted filename: Response to Reviewers.docx [file pone.0274428.s001.docx]

**Response to the reviewer #1’s comments**

We would like to extend our appreciation to the reviewer for the constructive comments and recommendations toward improving our manuscript. Your acknowledgement of the hard work and effort that went into the data collection, processing, and analysis, as well as the writing of the manuscript is greatly appreciated. All issues raised by the reviewer have been addressed and the manuscript has been updated accordingly. The following are our point-by-point responses to the reviewer’s comments:

Line by line comments [Line references indicate the location of the revised text and not the reviewer’s initial line]:

Line 66 -> changed in text, edited for clarity

Line 102-103 -> removed “comprehensive” as a descriptor and replaced it with “ecoregion-level”

Line 114 -> changed “State” to “state”

Line 115-116 -> removed “critical biophysical variables” and replaced it with the actual variables, “EVI, land surface albedo, and temperature”

Line 161-169 -> Added information to the caption in Figure 1 for clarity. It now more closely aligns with what is stated in the previous paragraph (143-147)

Table 1 -> In order to address the concern about the Cascades ecoregion only experiencing 6% burned area but still being included in the study, we added the total burned pixels and the percent burned area for the Marine West Coast Mountains and the North American Desert ecoregions to the text for reference (lines 143-147)

Line 188-191 -> rephrased the sentence without the use of “predominantly”, used suggested “prevailing” and “most substantial” instead

Line 210-212 -> Here we address the comment about EVI and its ability to differentiate between canopy and canopy background. EVI does not only report on canopy as the it says in the Reviewer comment. One of the main positive attributes of the data is its ability to distinguish the canopy from the canopy background. This is ideal for investigating the trajectory of EVI after fires of all severities, as changes to understory vegetation after low severity fires will still show up in the EVI averages.

Line 239-240 -> We clarified what time period the NBR was being calculated for in each year of the study period. The same early summer period (June 26 -August 12) was used for all biophysical variables in the study

Line 246 -> used the Reviewer comment to add a qualifying statement that low NBR may also represent low vegetation

Line 247 -> We made the decision to study the changes occurring within burned perimeters as areal averages instead of within pixels because we wanted to focus on the temporal aspect of biophysical change after wildfire. We are aware that some of the spatial heterogeneity of burn severity within fire perimeters was sacrificed when using MODIS data. However, MODIS has high temporal frequency and wide area coverage which makes it perfect for a temporal analysis of how EVI, temperature, and albedo change within burn perimeters throughout the five years after wildfire.

For our methodology, we are using the sum of all burned area for each year in our analysis. For example, 2003’s burned perimeters are used to get early-summer averages of EVI, albedo, and temperature in 2002 (prefire), and then in 2004, 2005, 2006, 2007, and 2008. This does away with the issue of growing wildfires over time in terms of total burned area because each year’s total burned area is considered. We also acknowledge that by using prefire NBR and postfire NBR that are taken a year apart, we are factoring in a year of recovery into the analysis of burn severity, EVI, albedo, and temperature. We believe that by using this methodology we can quantify the largest changes between a burned and unburned land surface. We plan on future studies where we investigate the spatial patterns within burn perimeters.

Line 247 Part 2 -> The area and number of burned pixels shown in Table 1 and 2 are meant to clue the readers into the general occurrence of wildfire in each ecoregion. These stats are meant to give the reader a sense of how much land has been burned in each ecoregion, as well as which ecoregions tend to burn the most. We are less concerned with the timing and number of fires that burn each year than we are with the mean climatology of the biophysical variables in the fire perimeters. Providing a detailed list of the number and timing of every fire that burned in California between 2003-2020 is outside the scope of the study. We are not trying to confuse the reviewer or the reader into thinking that the correlations shown later in the paper are calculated spatially, these correlations are calculated using areal averages within burned perimeters in the 7 different ecoregions.

Line 290 -> Early summer averages are an average for June 26 – August 12 for that variable within all the burned area within a given ecoregion. We take the average within the burned area one year prior and one to five years postfire (all the same burned area). Then all the prefire averages for that ecoregion are averaged, then all the one year, 2 year, etc postfire averages are averaged to get a single time series for each ecoregion for each variable (which are Figures 5, 6, 7). The Reviewer is correct in saying that EVI, albedo, and temperature averages are obtained by taking the average of each variable within a given years burned perimeter once a year for 5 years. See Line 247’s comment for more detail about the methodology.

Line 290 -> Fires in 2020 were considered – 2020 was the last year in which burned area perimeters were considered. We did not require that 5 years of postfire data be available in order for us to include the years 2016-2020. That means that 2016 includes 5 years of postfire data (because we use MODIS data for postfire analysis through 2021, but not burned area data in 2021), 2017 includes 4 years of postfire data, and 2018 includes 3 years, etc. Our study’s findings are limited by the satellite data temporal availability (2002-2021), and future changes in fire and climate regime associated with global warming may impact our findings. In order for us to consider the greatest number of years possible, we chose to use burned area data through 2020, even though it meant only considering one year post-fire for that year.

- We added a clarifying statement addressing this issue to the paper in Line 293-295

Table 2 -> The +/- values are the standard deviation of the mean values within ecoregions. I added this information to the Table 2 legend. We appreciate the reviewers comment about including the number of fires, however, we believe that because we are taking an average of each variable within burn perimeters/ecoregions, the total number of burned pixels from which these means/standard deviations are calculated is more beneficial to the reader.

Line 331 -> Reviewer may refer to Figure 3 and Lines 371-379 (and 361-364) for information about which years in the study area burned the most. Years 2018 and 2020 were certainly outliers in terms of total area burned in California. An investigative look at the annual summertime temperatures and humidity within each ecoregion is outside the scope of the project. However, some future work of the corresponding author does plan to look at both historical and projected fire weather within these same ecoregions.

Line 382 -> added Figure 4 to the text to address the problem of referring to something that did not exist.

Line 380 -> Refer to previous Lines 266-274 (methods section) for how the burn severity threshold were derived.

Line 382 -> The reviewer is correct in stating that the size of the fire does not affect the postfire averages because the time series is calculated by taking the average of each variable within the burned perimeter. Because all the prefire years, one year, two year, etc points are averaged together to obtain the final time series, the size of the fire (regardless of when the fire occurred – at the beginning or end of the time series) will not affect the final averages (it will just be a product of more points being averaged together, relative to other ecoregions that may have experienced fewer burn points).

Line 402 -> The methodology for obtaining the burn severity thresholds (Figure 4) uses the cumulative distribution of RdNBR values within pixels. These thresholds are defined based on the total number of burned pixels within each ecoregion, and not on the size of the ecoregion itself. For example, Figure 4 shows the reader that even though Klamath is not the second largest ecoregion, it has the second most occurrence of moderate to high severity burn pixels. In this way the reader can see that the Klamath and the Sierra Nevada are prone to higher severity burns, even though they are not necessarily the largest ecoregions.

Line 402 -> Thank you for pointing out that a definition of vegetation recovery was missing from the paper. We added a definition to the methods section where we describe the EVI datasets (Lines 212-214)

Table 1 -> We made the decision to combine the two land cover categories because they contain a similar “fractional coverage of woody vegetation”. This reasoning was also found in:

Jin Y, Randerson JT, Goetz SJ, Beck PSA, Loranty MM, Goulden ML. The influence of burn severity on postfire vegetation recovery and albedo change during early succession in North American boreal forests. Journal of Geophysical Research: Biogeosciences,. 2012; 117, G01036. <https://doi.org/10.1029/2011JG001886>.

Line 417 -> On line 421 we state that high correlations between RdNBR and EVI are expected because they are both sensitive to infrared reflectance. We have opted to follow the reviewer’s suggestion and remove the lines about how NBR is a control on RdNBR. Thank you.

Table 4 -> We have followed the Reviewer’s suggestions and labeled the x axis, added information to the caption and specified what is being regressed in the table.

Line 456 -> We added what is meant by vegetation recovery to the methods section after reading a previous comment. We removed part of a sentence that says that EVI return to prefire values was not found in any ecoregion “which is to be expected after such a brief interval”. We agree with the reviewer that this could be misleading to the reader in its implications.

Line 493 -> We have changed the wording from “significantly” to “substantially” following the reviewer’s suggestion

Line 507 -> We decided to remove the wording of “does not vary significantly” entirely to avoid confusion about the magnitude of that 1.9K change in prefire LST

Line 520 -> It is unclear which regressions the reviewer is referring to at this point in the text, but the answer to their question is that average LST was calculated within burned perimeters within each ecoregion. Land cover averages were only calculated for the initial tables and are not part of the later analysis, apart from the RdNBR cumulative frequency distributions (Results)

Line 547 -> changed the wording, removed Pacific Northwest and replaced with Klamath as suggested

Line 549 -> removed the word “pattern” as suggested

Line 572 -> added a statement that helps the reader understand what NDVI is in this context
Line 575 -> replaced “Furthermore” with “Further” as suggested

Line 583-584 -> foundational literature including Miller and Thode (2007) refer to complete mortality (high severity fire) of all live vegetation as “stand replacing”, regardless of type. This sentence is informing the reader on literature and is not necessarily about our findings, and we do not use the wording here to imply a forest ecosystem.

Line 620 -> added a citation, 64

Line 646 -> We appreciate that, and all of your comments. Thank you so much for your time.

Figure captions -> we went through the text and added information to figure captions as recommended by the reviewer
